# Earthworms Mitigate Pesticide Effects on Soil Microbial Activities

**DOI:** 10.3389/fmicb.2019.01535

**Published:** 2019-07-03

**Authors:** Sylvain Bart, Céline Pelosi, Alexandre Barraud, Alexandre R. R. Péry, Nathalie Cheviron, Virginie Grondin, Christian Mougin, Olivier Crouzet

**Affiliations:** UMR ECOSYS, INRA, AgroParisTech, Université Paris-Saclay, Versailles, France

**Keywords:** Lumbricidae, fungicide, enzyme activity, nitrification, ecotoxicology

## Abstract

Earthworms act synergistically with microorganisms in soils. They are ecosystem engineers involved in soil organic matter degradation and nutrient cycling, leading to the modulation of resource availability for all soil organisms. Using a soil microcosm approach, we aimed to assess the influence of the earthworm *Aporrectodea caliginosa* on the response of soil microbial activities against two fungicides, i.e., Cuprafor Micro^®^ (copper oxychloride, a metal) and Swing^®^ Gold (epoxiconazole and dimoxystrobin, synthetic organic compounds). The potential nitrification activity (PNA) and soil enzyme activities (glucosidase, phosphatase, arylamidase, and urease) involved in biogeochemical cycling were measured at the end of the incubation period, together with earthworm biomass. Two common indices of the soil biochemistry were used to aggregate the response of the soil microbial functioning: the geometric mean (Gmean) and the Soil Quality Index (SQI). At the end of the experiment, the earthworm biomass was not impacted by the fungicide treatments. Overall, in the earthworm-free soil microcosms, the two fungicides significantly increased several soil enzyme and nitrification activities, leading to a higher GMean index as compared to the non-treated control soils. The microbial activity responses depended on the type of activity (nitrification was the most sensitive one), on the fungicide (Swing^®^ Gold or Cuprafor Micro^®^), and on the doses. The SQI indices revealed higher effects of both fungicides on the soil microbial activity in the absence of earthworms. The presence of earthworms enhanced all soil microbial activities in both the control and fungicide-contaminated soils. Moreover, the magnitude of the fungicide impact, integrated through the SQI index, was mitigated by the presence of earthworms, conferring a higher stability of microbial functional diversity. Our results highlight the importance of biotic interactions in the response of indicators of soil functioning (i.e., microbial activity) to pesticides.

## Introduction

Earthworms and microorganisms represent the largest part of the living biomass in soils. They ensure a wide range of essential soil functions (Brown et al., 2000) and thus contribute to ecosystem services (Blouin et al., 2013; Bertrand et al., 2015). As ecosystem engineers (Jones et al., 1994), earthworms play key roles in the dynamic of the soil organic matter (SOM) and of the resource availability for other soil organisms through tight interactions with microorganisms, which act as chemical engineers (Scheu, 1987; Edwards and Fletcher, 1988). The mechanical and biological activities of earthworms catalyze SOM decomposition, carbon and nitrogen mineralization, and nutrient turnover, by modulating microbial biomass and activity (Daniel and Anderson, 1992; Zhang and Hendrix, 1995; Tiunov and Scheu, 1999). Different earthworm species are known to increase soil microbial respiration (Scheu, 1987) or soil enzyme activities related to C, N, and P cycling (Tao et al., 2009; Dempsey et al., 2013), especially in the drilosphere (casts and burrow walls, Loquet et al., 1977; Aira et al., 2003). These works highlight significant effects of earthworms on the abundance of various microbial groups (i.e., ammonifiers, denitrifiers, and proteolytic bacteria). The mucus produced by earthworms is also a nutrient resource for microbial activity (Martin et al., 1987). However, by ingesting microbial biomass, they can also decrease the total microbial biomass while increasing the specific activities of its residual component (i.e., extra-cellular enzyme activities, Zhang et al., 2000; Aira et al., 2009).

The use of pesticides in agroecosystems may impair biodiversity and biological activity in cultivated soils (Bengtsson et al., 2005; Hole et al., 2005). The normalization of experimental conditions to assess pesticide impacts on soil organisms greatly contributed to such a historical separation between biological models, while ecological approaches spoke up for taking into account biotic interactions in the study of ecosystem function under chemical stress (Burrows and Edwards, 2004; Clements and Rohr, 2009). In turn, few investigations on the effects of pesticides on soil biological functioning have considered the fundamental interaction between the soil fauna (earthworms) and microorganisms, as earthworms can increase microbial activity, even in insecticide-contaminated soils (Sanchez-Hernandez et al., 2018).

Earthworms are often used as soil biological indicators (Spurgeon et al., 2003), and the impacts of pesticides on earthworms have been extensively documented (Pelosi et al., 2014). These physical ecosystem engineers continuously modify soil microhabitats, and thereby influence on microbial life and the related biogeochemical activities. Pesticide application can also impact soil microbial communities and their activity, but with much more contrasted results (by decreasing or increasing them) depending on the active compound and the microbial groups (Chen et al., 2001; Wainwright, 2006; Niemi et al., 2009), with possible outcomes for microbe-mediated processes (Muñoz-Leoz et al., 2013). In this respect, enzyme activities are useful indicators of soil health because enzymes contribute to nutrient cycling (Burns and Dick, 2002) and their activity can be used as a proxy of changes in soil functioning due to the alteration of microbial communities in response to heavy metal exposure (Kandeler et al., 1996; Speir and Ross, 2002). The multiple direct and indirect effects jointly affect enzyme activity, which results in an increase, decrease or leveling off of its catalytic activity (Gianfreda and Rao, 2008; Riah et al., 2014). Copper (Cu) can be used as an inorganic pesticide in organic farming. Like other metals, depending on its concentrations, copper can be an oligo-element acting as a co-factor for some enzymes, or a toxicant for the cellular activities (Giller et al., 2009). Chemical stressors can affect narrow niche functions (N_2_ fixation or nitrification) more than broad-scale niche processes (enzyme activities), which may display higher diversity and functional redundancy (Pell et al., 1998; Crouzet et al., 2016; Karas et al., 2018).

There is a general knowledge gap about the relationship between taxonomic diversity and ecosystem functions, so that functional rather than taxonomic diversity could be more suitable to investigate microbial roles in ecosystems (Zak et al., 1994). Microbial functional diversity is defined as the numbers, types, activities, and rates at which a range of substrates is metabolized by the microbial communities to contribute to ecosystem processes (e.g., organic matter mineralization, Zak et al., 1994). To assess the impact of soil contamination on soil functions, several indices were developed as indicators of soil quality by aggregating different soil microbial activities, especially enzymatic activities involved in biogeochemical cycles (Bending et al., 2002; Rodríguez-Loinaz et al., 2008). The geometric mean (Gmean) index can be a suitable proxy of soil functional diversity (Lessard et al., 2014), since it was calculated with a sufficient range of activities depending on numerous metabolic reactions and interactions among members of the soil biota (Nannipieri et al., 2002). The SQI is another index that characterizes changes in the measured microbial activity (decrease of increase) following a treatment (Bloem et al., 2006).

The aim of our work was to quantify the potential benefit of the presence of earthworms for the tolerance of the soil microbial community to fungicides. It was based on the assumption that earthworms can modulate microbial activity and exposure to contaminants due to their ecosystem engineer role. The hypotheses were that (i) the two fungicides would differently impact microbial activity due to their different fates in the soil, and (ii) earthworms would confer a higher tolerance to the microbial communities exposed to fungicides. We carried out a dose-effect study in soil microcosms to assess the influence of the presence of earthworms on the impact of two commercial formulations of fungicides (Cuprafor Micro^®^ with copper oxychloride as the active ingredient, and Swing^®^ Gold with epoxiconazole and dimoxystrobin as active ingredients) on soil microbial activities. These fungicides were selected because they can affect both earthworms (Pelosi et al., 2014) and soil microorganisms at doses close to recommended application rates, whereas herbicides or insecticides usually disturb microbial processes at much higher doses (Wainwright, 2006; Muñoz-Leoz et al., 2013). In addition, the inorganic copper-based fungicide does not dissipate, while the synthetic organic fungicide does. To assess the fungicide effects, some earthworm endpoints were measured (survival and biomass) and the microbial responses targeted several microbial activities involved in biogeochemical cycling and the whole functional microbial diversity (with enzyme indexes).

## Materials and Methods

### Soil and Earthworms

The soil used for all experiments was sampled from the top 0–20 cm in a permanent grassland in Versailles (48°48′ N, 2°5′ E) where no chemical had been applied for more than 20 years. It was a Luvisol (FAO soil classification) and its main physical characteristics were as follows: pH 7.5, organic matter 32.6 g kg^−1^, C/N 12.7, 29% sand, 48% silt, 23% clay, and 25.2 mg Cu kg^−1^ (see Bart et al., 2017 for more details). The soil was air-dried and sieved to 2 mm.

Mature *Aporrectodea caliginosa* s.s individuals were collected by hand-sorting from an agricultural field in Estrées-Mons (49°52′ N 3°01′E). Their weight ranged from 600 to 1 000 mg. They were stored in the soil used for the experiments at 15 ± 1°C, 24 h darkness for at least 10 days before the experiments.

### Pesticides

Swing^®^ Gold (BASF Agro SAS, dimoxystrobin 133 g L^−1^, epoxiconazole 50 g L^−1^) is an organic synthetic fungicide widely used in conventional farming to protect cereal crops. The Recommended Dose (RD) was calculated as 1.16 10^−3^ mL kg^−1^ (corresponding to 150 μg kg^−1^ of dimoxystrobin and to 60 μg kg^−1^ of epoxiconazole) of dry soil for a soil density of 1.29 and considering that the active compounds of this fungicide are mostly found in the top 10 cm of soil (McDonald et al., 2013; Chabauty et al., 2016). Based on the LC_50_ estimated to be 6.3 times the RD for *A. caliginosa* (Bart et al., 2017), we tested 0.33, 1, and 3 times the RD of this commercial formulation.

Cuprafor Micro^®^ (Quimicas del Valles, 50% copper oxychloride) is a metal-based fungicide commonly used in organic farming to prevent spore germination; it is authorized in organic management. The RD was calculated as 15.5 mg kg^−1^ (corresponding to 7.75 mg Cu kg^−1^ of dry soil) for a soil density of 1.29 and considering that the active compounds of this fungicide are mostly found in the top 5 cm of soil (Couto et al., 2015). Based on literature reviews (Ma, 1984; Spurgeon et al., 2004; Bart et al., 2017; Pesticide Properties DataBase [PPDB], 2018) and taking into account that Cu can accumulate in soils, we tested 3.33, 10, and 30 times the RD, which corresponds to the addition of 25.8, 77.5, and 232.3 mg kg^−1^ of Cu.

### Experimental Design

Soil microcosms were built up with five replicates for each condition. Each microcosm corresponded to a 1-L plastic vessel with a removable perforated cover for gas exchange. Each vessel contained 500 g of dry soil and 24 g of dry horse dung as a food resource for earthworms, corresponding to a feeding of 6 g ind^−1^ month^−1^ as suggested in Lowe and Butt (2005) and in Bart et al. (2018) for *A. caliginosa*. The soil moisture was adjusted to 70% of the maximum water holding capacity (mWHC) using the fungicide solutions or tap water as controls. The food moisture was also adjusted to 70% of mWHC and mixed with the soil. Four *A. caliginosa* individuals were introduced in each vessel, and incubation was run for 28 days in a climate-controlled room at 15 ± 1°C. Earthworms were weighed on day 0 and at the end of the experiment (on day 28). The soil moisture content was controlled once a week. A similar set of microcosms was set up without adding earthworms.

### Microbial Activity

All soil enzyme activities mainly come from microorganisms (prokaryotes and fungi) and are involved in the nutrient cycles of carbon (C), nitrogen (N), and phosphorus (P) (Burns et al., 2013), through organic matter mineralization. Nitrification is a key step of nitrogen cycling ensured by specific bacterial and archaeal guilds (Prosser, 2005), which are known to be highly sensitive to pesticides (Crouzet et al., 2016). The potential nitrification activity (PNA) and soil enzymes β-D-glucosidase (GLU), phosphatase (PHOS), urease (URE), and arylamidase (ARM) were assessed in each microcosm on day 28 after the fungicide treatments. Three analytical replicates were measured for each microcosm and each activity.

PNA was determined in accordance with Petersen et al. (2012), with some modifications specified in Corbel et al. (2015). Briefly, 4 g of fresh soil were sampled and mixed with 25 mL of MilliQ water and (NH_4_)_2_SO_4_ at a final concentration of 1 mM. Samples were incubated at 25°C under continuous shaking (150 rpm). After 2.5 and 45 h, 1 mL was sampled and centrifuged at 13,000 g for 5 min, and supernatants were stored at −20°C until analyses of the nitrate (N0_3_^−^) and nitrite (NO_2_^−^) ions by colorimetry according to the Griess reaction. The supernatants were dropped in microplates and the Griess solution was added (HCl, 0.5M, vanadium chloride III (Sigma-Aldrich 208272) at 1 g L^−1^, sulfanilamide (Sigma-Aldrich S9251) at 2.5 g L^−1^ and N-(1-naphthyl)-ethylenediaminedihydrochloride (Sigma-Aldrich 222488) at 0.25 g L^−1^), and then incubated at 60°C for 1.5 h. The optical densities were determined at 540 nm with a microplate reader (SAFAS Xenius, Monaco). Results were then expressed as PNA, which is the rate of N-NO_3_^−^ + N-NO_2_^−^ production during activity measurements (for 45 h), in μg N _released_ g^−1^ h^−1^.

The soil enzymes β-D-GLU, PHOS, ARN, and URE were measured according to the ISO 20130 (2018) standard, with a slight modification for URE. All measurements were performed at the soil pH, in an unbuffered soil water solution, in accordance with Lessard et al. (2013). Three aliquots of 4 g of fresh soil each were sampled in each microcosm, and each one was mixed with 25 mL of MilliQ water (10 min, ambient temperature, continuous shaking at 250 rpm). Aliquots of soil solution (125 μL) were incubated in 96-well microtiter plates with the following substrates: 4-nitrophenyl-β-D-glucopyranoside (final concentration in the wells: 8.3 mM, incubation time: 1 h at 37°C) for GLU, 4-nitrophenylphosphate (8.3 mM, 30 min at 37°C) for PHOS, urea (80 mM, 3 h at 25°C) for URE, and L-leucine β-naphthylamide-hydrochloride (1.3 mM, 1 h at 37°C) for ARYL-N. Each substrate was added at a concentration corresponding to its saturating concentration. After the incubation period, reactions were stopped by adding a CaCl_2_ solution (0.5 M) and Tris–HCl (0.1 M, pH 12) for GLU, PHOS, and URE. For ARM, reactions were stopped with ethanol 96% (*v/v*). The microplate was then centrifuged at 3,000 g for 5 min, and an aliquot of 0.2 mL from each well was used to evaluate enzyme activity. The para-nitrophenol (pNP) released by GLU and PHOS activities was measured at 405 nm, and the β-naphthylamine released by ARYL-N activity was determined at 540 nm, using a microplate reader (SAFAS, Monaco). Enzyme activities were calculated based on external calibration curves using standards (Sigma): p-nitrophenol (final concentrations in the wells ranged from 0 to 0.4 mM), β-naphthylamine (from 0 to 0.2 mM). The ammonium ion NH_4_^+^ released by URE activity was determined at 610 nm with an HACH reagent (Loveland, CO, United States), and enzyme activity was calculated based on a calibration curve using an NH_4_Cl standard (Sigma), with final concentrations in the wells ranging from 0 to 0.3 mM NH_4_^+^. Calibration curves were performed in similar reaction mixtures as each enzyme but without soil solution, since no difference in adsorption of pNP and β-naphthylamine standards was expected in such similar soil samples (the same soil and amount of OM in all microcosms). Results were then expressed in mU g^−1^ dry soil, representing nanomoles of product released per minute and per g of equivalent dry soil.

### Soil Functional Diversity

The impact of pesticides on soil microbial functioning was assessed using the GMean index (Hinojosa et al., 2004), which aggregates each of the individual microbial activities. The Gmean index is considered as a suitable proxy of functional microbial diversity (Lessard et al., 2014):

$$\text{GMean}={\left(\prod_{i=1}^{n}{}_{y_{i}}\right)}^{1/n}$$

where *y_i_* is the enzyme activity or PNA, *n* is the number of soil enzymes and the PNA (5). High GMean values mean high microbial functional diversity (Lessard et al., 2014).

The second index was the SQI, as described by Bloem et al. (2006). It was calculated using the average factorial deviation from the reference value (Ten Brink et al., 1991):

$$\text{SQI}=10^{\mathrm{logm}-\frac{\sum_{i=1}^{n}\left|\mathrm{logm}-{\mathrm{logn}}_{i}\right|}{n}}$$

where *m* is the reference soil (mean value of enzyme activity or PNA in the control soil in the presence or in the absence of earthworms, set to 100%), and *n* are the measured values as percentages of the reference soil. A decrease of the SQI highlights a modification (increase and/or decrease) in the soil microbial activity.

### Data Analysis

All statistical analyses were performed using R software Core Team (2015). The analyses of biomass changes between day 0 and day 28, and the assessment of the mortality rate were performed using the non-parametric Wilcoxon signed-rank test. When the normality and homoscedasticity conditions were satisfied, each microbial activity and index was analyzed using a two-way ANOVA to test the effect of fungicide concentrations, of the presence of earthworms, and of the interaction between these two factors. Then, a Tukey test was performed to assess the difference between pesticide treatments in the soils in the presence or in the absence of earthworms. When the normality and homoscedasticity conditions were not satisfied, the non-parametric kruskalmc (multiple comparison) test (adjusted *p*-values based on Bonferroni’s corrections were applied) to assess the difference between pesticide treatments in the soils in the presence or in the absence of earthworms. The percentages of increase of the PNA, Gmean index, and SQI between the control and the highest concentration tested were compared between the soils in the presence and in the absence of earthworms using the Wilcoxon signed-rank test.

## Results

### Effects of the Copper Fungicide

An earthworm mortality rate of 5% was recorded in the Cu3.33 and the control treatments at the end of the experiment (after 28 days). No mortality occurred in the Cu10 and Cu30 treatments. There was no significant difference in earthworm mortality or weight along the experiment whatever the Cu fungicide concentration tested.

The responses of enzyme activities and PNA in the control soil and Cu-treated soils in the presence or in the absence of earthworms are presented in Table 1. In the absence of earthworms, a significant effect of the Cu treatment was measured on three enzyme activities (GLU, PHOS, and ARN), and there were significant differences between the control and Cu treatments. Glucosidase activity significantly increased as compared to the control, only following the Cu3.33 treatment (Table 1). Phosphatase activity decreased by 25.1% in the Cu30 treatment as compared to the control. Arylamidase activity increased with increasing Cu concentrations, to reach +60% of the control value in the Cu30 treatment. The presence of earthworms significantly increased all enzyme activities as compared to the earthworm-free soils. It also resulted in a lower difference or no difference at all between enzyme activities in the control and Cu-treated soils. Then, only phosphatase activity significantly decreased by 22.7% in the Cu30 treatment as compared to the control. This decrease was of the same magnitude as the 25.1% observed in the earthworm-free soils.

**Table 1:** Enzyme activities (mU g^−1^ dry soil) and potential nitrification activity (PNA, μg NO_3_ g^−1^ dry soil) in a control soil and the same soil spiked with different concentrations of Cuprafor micro^®^ (3.33, 10, and 30 times the RD corresponding to 25.8, 77.5, and 232.5 mg kg^−1^ of copper, abbreviated Cu3.3, Cu10, and Cu30, respectively), in the presence or in the absence of earthworms (*n* = 5, ±SD).

	Treatment								Effects		
Enzyme and microbial activities	Earthworm absence				Earthworm presence						
	Control	Cu 3.33	Cu 10	Cu 30	Control	Cu 3.33	Cu 10	Cu 30	E	t	txE
β-D-glucosidase	12.5 ± 1.1a	16.4 ± 1.2b	13.1 ± 0.7a	14.3 ± 0.8ab	25.0 ± 1.4A	24.3 ± 1.7A	23.9 ± 3.1A	25.2 ± 1.0A	^∗∗∗^	^∗^	^∗^
Phosphatase	20.7 ± 1.3a	20.2 ± 1.4a	17.1 ± 1.4ab	15.5 ± 1.6b	34.8 ± 2.7A	34.9 ± 2.6A	30.8 ± 3.9B	26.9 ± 1.0C	^∗∗∗^	^∗∗∗^	ns
Urease	17.6 ± 2.8a	21.3 ± 2.6a	19.7 ± 1.4a	20.5 ± 2.4a	30.0 ± 2.1A	31.9 ± 2.0A	29.7 ± 2.6A	28.3 ± 2.1A	^∗∗∗^	ns	ns
Arylamidase	6.3 ± 0.4a	8.2 ± 0.6b	8.9 ± 0.3bc	10.1 ± 1.4c	13.1 ± 0.7AB	12.4 ± 0.4A	12.7 ± 1.1AB	13.7 ± 0.5B	^∗∗∗^	^∗∗∗^	^∗∗∗^
PNA	0.46 ± 0.05a	0.62 ± 0.04b	0.64 ± 0.04b	0.71 ± 0.05c	0.66 ± 0.05AB	0.65 ± 0.05A	0.64 ± 0.04A	0.73 ± 0.04B	^∗∗∗^	^∗∗∗^	^∗∗∗^

*Different letters mean significant differences between treatments, and the result of the two-way ANOVA test is presented as follows: “t” is for treatment effect, “E” for earthworm effect and “(txE)” for their interaction. “ns” means no significant difference, ^∗^p < 0.05 and ^∗∗∗^p < 0.001.*

The two-way ANOVA test revealed that PNA was significantly affected by the presence of earthworms [*F*(1,32) = 23.5, *p* ≤ 0.0001], by the Cu treatments [*F*(3,32) = 25.4, *p* ≤ 0.0001], and by the interaction between these two factors [*F*(3,32) = 11, *p* ≤ 0.0001] (Table 1). Considering the earthworm-free soil, PNA significantly increased by +37, +40, and +57% in the Cu3.33, Cu10, and Cu30 treatments, respectively, as compared to the control. In the soils that harbored earthworms, statistical analyses did not reveal any effect whatever the Cu applications as compared to the control, but PNA was higher following the Cu30 treatment than following the Cu3.33 and Cu10 treatments. The magnitude of the PNA increase between the control and Cu30 treatment was much higher in the earthworm-free soils (57 ± 11%) than in the soils harboring earthworms (12 ± 5%) (Wilcoxon test, *p* = 0.012).

**FIGURE 1:**
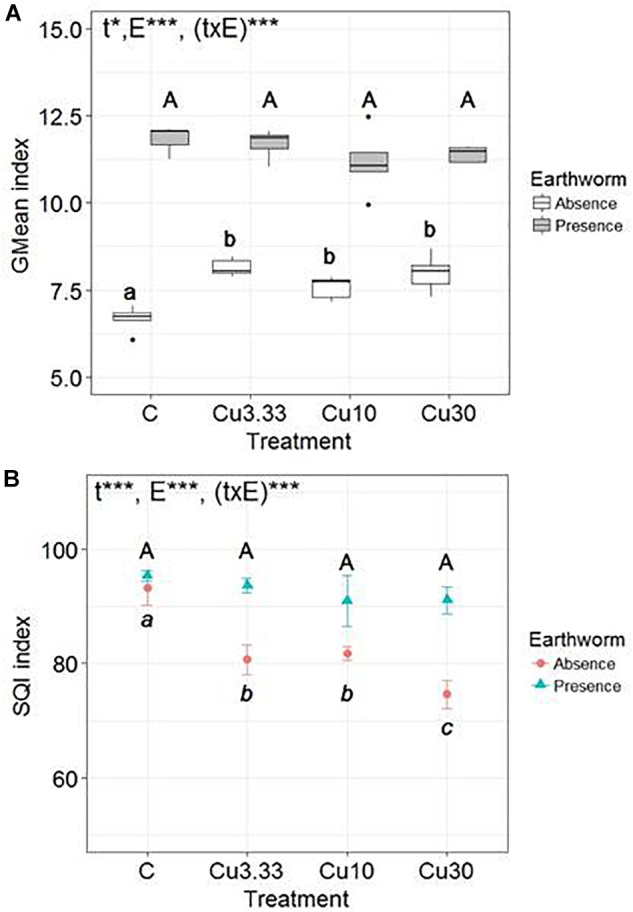
Box-plots of **(A)** the geometric mean (GMean index) and **(B)** the Mean values (*n* = 5, ±SD) of the Soil Quality Index (SQI) of microbial activities (β-D-glucosidase, phosphatase, urease and arylamidase, potential nitrification activity) in soils spiked with different Cuprafor Micro^®^ concentrations (3.33, 10, and 30 times the RD, corresponding to 25.8, 77.5, and 232.5 mg kg^−1^ of copper, abbreviated Cu3.3, Cu10, and Cu30, respectively), and a control, in the presence or in the absence of earthworms. Different letters mean significant differences between treatments and the results of the two-way ANOVA test are presented as follows: “t” is for treatment effect, “E” for earthworm effect, and “(txE)” for their interaction. “ns” means no significant difference, ^∗^*p* < 0.05 and ^∗∗∗^*p* < 0.001.

There was a significant effect, highlighted by the two-way ANOVA test, of the Cu treatments [*F*(3,32) = 4.2, *p* = 0.013], of earthworms [*F*(1,32) = 702, *p* ≤ 0.0001], and of the interaction between these two factors [*F*(3,32) = 7.8, *p* ≤ 0.0001] on the GMean index (Figure 1A). The presence of earthworms strongly promoted soil microbial activity in all treatments. When considering only the set of earthworm-free soil microcosms, the GMean index significantly increased with increasing Cu application rates (by 19.9 ± 7.9% in Cu30 as compared to the control), while there was no effect of the Cu treatment on the GMean index in the set of soil microcosms harboring earthworms.

The two-way ANOVA test revealed a significant effect of the Cu treatments [*F*(3,32) = 36, *p* ≤ 0.0001], of earthworms [*F*(1,32) = 161, *p* ≤ 0.0001], and of the interaction between these two factors [*F*(3,32) = 14, *p* ≤ 0.0001] on the SQI (Figure 1B). Significant effects of the Cu application rates on the SQI were only observed in the earthworm-free soils in which the SQI decreased by 20.0 ± 3% between the control and the Cu30 treatment.

### Effects of the Swing^®^ Gold fungicide

No earthworm mortality was recorded in the SG0.33 treatment. A mortality rate of 5% was found in the SG1 and control treatments, and 20% in the SG3 treatment. Nevertheless, these results were not statistically significant. No impact was recorded on the earthworm biomass.

The enzyme activity and PNA responses in the control soil and the soil treated with the SG fungicide, in the presence or in the absence of earthworms, are presented in Table 2. In the earthworm-free soil microcosms, the SG treatment had a significant effect on all enzyme activities, but there was no difference in phosphatase activity between the control and the SG3 treatment, contrary to the Cu treatments. GLU, URE, and ARN activities significantly increased by 25, 19, and 18%, respectively, in the SG3-treated soils as compared to the control. The presence of earthworms significantly increased all enzyme activities, as previously observed with the Cu treatment. Considering the set of soil microcosms harboring earthworms, a significant effect of the SG treatment was observed on phosphatase and urease activity between the control and the SG0.33 and SG3 treatments, respectively. Urease activity increased by 17.0% in the SG3 treatment as compared to control.

**Table 2:** Enzyme activities (mU g^−1^ dry soil) and potential nitrification activity (PNA, μg NO_3_ g^−1^ dry soil) in a control soil and the same soil spiked with different Swing^®^ Gold concentrations (0.33, 1, and 3 times the RD abbreviated SG0.33, SG1, and SG3, respectively), in the presence or in the absence of earthworms (*n* = 5, ±SD).

	Treatment								Effects		
Enzyme and microbial activities	Earthworm absence				Earthworm presence						
	Control	SG 0.33	SG 1	SG 3	Control	SG 0.33	SG 1	SG 3	E	t	t xE
β-D-glucosidase	12.5 ± 1.1a	13.6 ± 0.7a	12.3 ± 1.2a	16.6 ± 1.6b	25.0 ± 1.4A	25.5 ± 1.1A	24.8 ± 1.6A	23.7 ± 0.6A	^∗∗∗^	^∗^	^∗∗∗^
Phosphatase	20.7 ± 1.3ab	23.5 ± 2.1a	20.2 ± 0.7b	20.3 ± 2.4ab	34.8 ± 2.7A	39.3 ± 1.6B	38.0 ± 1.4AB	38.0 ± 2.1AB	^∗∗∗^	^∗∗^	ns
Urease	17.6 ± 2.8a	19.1 ± 2.5ab	18.7 ± 2.0ab	21.5 ± 0.6b	30.1 ± 2.1A	32.3 ± 0.9AB	33.5 ± 2.1AB	35.2 ± 1.2B	^∗∗∗^	^∗∗∗^	ns
Arylamidase	6.3 ± 0.4a	7.7 ± 1.3b	7.4 ± 0.5ab	7.8 ± 0.3b	13.6 ± 0.7A	12.8 ± 0.7A	13.1 ± 0.5A	12.8 ± 0.5A	^∗∗∗^	ns	^∗∗^
PNA	0.46 ± 0.05a	0.66 ± 0.06b	0.63 ± 0.04b	0.79 ± 0.05c	0.66 ± 0.04A	0.76 ± 0.05B	0.76 ± 0.05BC	0.87 ± 0.05C	^∗∗∗^	^∗∗∗^	ns

*Different letters mean significant differences between treatments, and the result of the two-way ANOVA test is presented as follows: “t” is for treatment effect, “E” for earthworm effect, and “(txE)” for their interaction. “ns” means no significant difference, ^∗^p < 0.05 and ^∗∗∗^p < 0.001.*

The two-way ANOVA test revealed that PNA was significantly affected by the presence of earthworms [*F*(1,32) = 68, *p* ≤ 0.0001] and the SG treatments [*F*(3,32) = 52, *p* ≤ 0.0001], but not by their interaction [*F*(3,32) = 2.9, *p* = 0.05] (Table 2). PNA increased along with the increase in SG application rates, in the presence or absence of earthworms. However, the magnitude of the increase in PNA between the control and SG3-treated soils was much higher in the earthworm-free soils (73.4 ± 11.4%) than in the soils harboring earthworms (32.4 ± 8.2%) (Wilcoxon test, *p* = 0.008).

**FIGURE 2:**
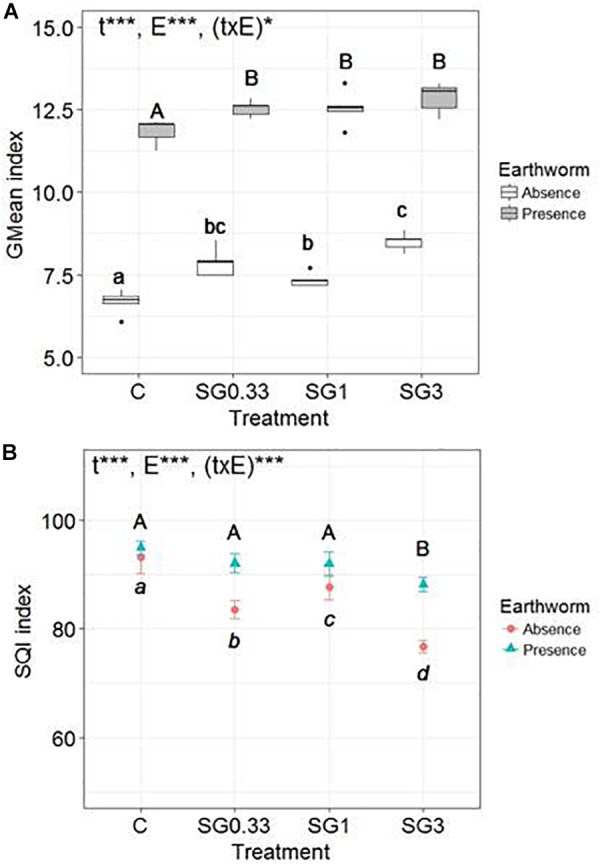
Box-plots of **(A)** the geometric mean (GMean index) and **(B)** the Mean values (*n* = 5, ±SD) of the Soil Quality Index (SQI) of microbial activities (β-D-glucosidase, phosphatase, urease and arylamidase, potential nitrification activity) in soils spiked with different Swing^®^ Gold concentrations (0.33, 1, and 3 times the RD abbreviated SG0.33, SG1 and SG3, respectively) and a control soil, in the presence or in the absence of earthworms. Different letters mean significant differences between treatments, and the result of the two-way ANOVA test is presented as follows: “t” is for treatment effect, “E” for earthworm effect, and “(txE)” for their interaction. “ns” means no significant difference, ^∗^*p* < 0.05 and ^∗∗∗^*p* < 0.001.

There was a significant effect, highlighted by the two-way ANOVA test, of the SG treatments [*F*(3,32) = 25, *p* ≤ 0.0001], of earthworms [*F*(1,32) = 1,681, *p* ≤ 0.0001], and of the interaction between these two factors [*F*(3,32) = 3, *p* = 0.045] on the GMean index (Figure 2A). The presence of earthworms promoted overall soil microbial activity in all modalities. There was a significant increase of the GMean index with the increase in SG application rates, in the absence or in the presence of earthworms. However, the magnitude of the GMean increase between the control and the SG3 treatment was significantly higher in the absence of earthworms (+27.4 ± 4.0%) than in their presence (+8.6 ± 3.9%) (Wilcoxon test, *p* = 0.008).

The two-way ANOVA test revealed a significant effect of the SG treatments [*F*(3,32) = 61, *p* ≤ 0.0001], of earthworms [*F*(1,32) = 109, *p* ≤ 0.0001], and of the interaction between these two factors [*F*(3,32) = 11.9, *p* ≤ 0.0001] on the SQI (Figure 2B). There was a significant effect of the different SG application rates on the SQI, in the absence or in the presence of earthworms. However, the magnitude of the SQI decrease between the control and the SG3 treatment was significantly higher in the absence of earthworms (−17.7 ± 1.3%) than in the presence of earthworms (−7.1 ± 1.4%) (Wilcoxon test, *p* = 0.008).

## Discussion

The absence of an impact of fungicides on earthworm biomass and mortality during the experiment, whatever the concentrations applied, validates the sublethal concentration values retained for this experiment. Besides, no dormancy was observed in the earthworms collected at the end of the experiment. Nevertheless, the absence of an effect on biomass did not inform on possible impacts on earthworm behavior in the soils containing pesticides (Capowiez et al., 2006; Dittbrenner et al., 2011). Therefore, possible effects of fungicides on earthworm burrowing or feeding activities, which are key parameters related to their influence on soil microbial communities (Edwards and Bohlen, 1996), cannot be excluded.

### Impact of Fungicides on Soil Microbial Activity in the Absence of Earthworms

In the absence of earthworms, contrasted responses were reported depending on the microbial activities and the fungicides, but overall both commercial fungicides increased the whole soil microbial activity, integrated with the GMean index. The decrease in phosphatase activity with increasing application rates of the Cu-based fungicide is in accordance with several previous works showing that phosphatase activity decreased in soils treated with 150 and 450 mg Cu kg^−1^ (Wyszkowska and Wyszkowski, 2010). Urease activity remained stable in our experiment, even at the highest concentration tested (232.5 mg kg^−1^ of Cu), similarly to previous works where Cu hydroxide or CuCl_2_ did not impact urease activity even at 156 mg kg^−1^ (Wightwick et al., 2013). Conversely, Nor (1982) reported thorough inhibition of soil urease at 120 mg Cu kg^−1^. A theoretical PNEC for metals in soils can be predicted for given soil properties, on the basis of the HC 5% hazardous effect derived from SSD analyses (including plants, the meso- and macrofauna, and microorganisms) computed with literature data collection (Oorts et al., 2006b; Smolders et al., 2009). For our soil properties, the PNEC value was around 78.5 mg Cu kg^−1^ dry soil, resulting in expected toxic effects on some microbial activities at Cu30 (232 mg kg^−1^). The increases in GLU, ARM, and PNA activity in the Cu-treated soils as compared to the controls were not expected. Copper has indeed been observed to impair soil microbial biomass or activity (Giller et al., 2009), more so following spiking of solutions of metal salts (Oorts et al., 2006a; Smolders et al., 2009). The impairment of microbial enzyme activity by metals might result from cellular toxicity that decreases the whole metabolism, or from the reaction of metal ions with the substrate or the protein-active groups of enzymes in soils (Deng and Tabatabai, 1995).

Two main hypotheses could explain such differences between the stimulation of microbial activity observed in our experiment (in the presence or in the absence of earthworms) and the numerous previous works underlining toxic effects of Cu (inhibition) at doses similar to those tested in this work. First, the addition of horse manure (at 4.8% w/w dry soil) provided a very high level of organic matter which strongly adsorbed copper ions, and likely decreased copper bioavailability for the same total input as compared to the previous cited literature. Such metal buffering by OM addition may have alleviated the Cu toxicity to soil microbial activities in our experiment. Second, another essential difference was that almost all these studies investigated impacts of Cu salt solutions, while we used a commercial formulation of Cu oxide containing unknown adjuvants and surfactants. These compounds can deeply modify the fate of copper in the soil and its effects on soil microorganisms. Adjuvants of commercial formulations of pesticides might act as available sources of nitrogen and carbon able to stimulate microbial biomass and activity (Crouzet et al., 2010; Mijangos et al., 2010). As a result, in our experimental conditions, the microbial exposure to copper would be below toxic thresholds, which would be consistent with the response patterns of microbial activities showing a hormetic-like response. Some previous findings been already observed for several soil microbial enzymes or nitrification in response to metal stress (Langdon et al., 2014; Han et al., 2019). Overcompensation in response to disruption in homeostasis was assumed the fundamental mechanism of hormesis, existing to preserve organism homeostasis (Calabrese and Baldwin, 2002).

Regarding the SG fungicide, an experiment with a commercial fungicide containing the same active substances (dimoxystrobin and epoxiconazole) showed negative effects of these fungicides on the activity of soil dehydrogenase and urease recorded only at 100-fold the recommended field rates (Jastrzȩbska and Kucharski, 2007). Along with ours, these results underline that, at realistic doses, fungicides based on a dimoxystrobin – epoxiconazole mixture do not negatively affect the related soil enzyme activities (i.e., PHOS, GLU, URE, and ARN), but could disturb soil nitrification (PNA). Overall, the increases of several microbial activities have already been observed with other organic synthetic pesticides (glyphosate, Haney et al., 2002; carbendazim, tebuconazole, and captan, Burrows and Edwards, 2000; Chen et al., 2001; Cycoń et al., 2006). A first assumption about this phenomenon is that the fungicides killed or inhibited the activity of certain groups of non-target fungi. On the short term, dead fungal biomass might be used as a food resource by living microorganisms, and this could lead to greater bacterial activity, along with decreased competition for other resources (Monkiedje et al., 2007). Another assumption is that fungicides kill or inhibit the soil microfauna, such as protozoa or nematodes which are predatory for microorganisms (Ekelund and Rønn, 1994; Ronn et al., 2002), thus turning off the top-down regulation of microbial biomass and activity. Finally, as regards copper, we used commercial formulations instead of pure active compounds. The surfactants and the adjuvants contained in commercial products may influence the impacts of active ingredients on microbial activity (Crouzet et al., 2010).

### Earthworms Shape the Responses of Microbial Activity to Fungicides

One of the important results of this study is that the presence of earthworms increased all microbial (soil enzyme and nitrification) activities, even in the fungicide-treated soils. Overall, this result could lead us to think that earthworm behavior was not impaired in the SG- or Cu-treated soils, as earthworm effects on the different activities were similar in all soils. The stimulation of microbial activity by earthworms has already been observed (Scheu, 1987; Binet et al., 1998; Aira et al., 2003; Mougin et al., 2013). However, our study, along with that of Sanchez-Hernandez et al. (2018), is the first to show that this ability was preserved in pesticide-treated soils, while earthworm-free soils were disturbed. The absence of a pesticide effect on the earthworms, at the doses tested in our experiment, may have contributed to the conservation of their benefits for microbial activity. The higher tolerance and stability of the activity and functional diversity of microbial communities in response to pesticides conferred by the presence of earthworms could be explained by the ecosystem engineer role of earthworms that provides favorable micro-habitats for microbial communities (Haynes et al., 2003; Lipiec et al., 2016). Even if we did not assess the dynamic of exposure to the two pesticides, earthworm activity probably modified microorganism exposure to copper or organic fungicides. Earthworms can indeed influence the fate of metals or organic pesticides in soils (Sizmur and Hodson, 2009; Rodriguez-Campos et al., 2014).

### Suitability of Microbial Activity Endpoints

Pesticide effects seemed to depend on the microbial metabolism underlying the measured activity. In our study, the magnitude of the effects on nitrification was higher than on the various soil enzyme activities. This might be explained by the fact that PNA measured the activity of physiologically active and viable microorganisms, while the measure of soil enzyme activities captures intracellular and extracellular activities. A significant amount of hydrolytic activity comes from extracellular (abiotic) enzymes bound and protected by soil colloids (Knight and Dick, 2004); they do not require the intracellular integrity of microbial cells to be expressed (Burns and Dick, 2002). Thus, it has been evidenced that decreases in activity in response to soil management are reflected more by the activity of extracellular stabilized enzymes than by enzymes belonging to viable microbial cells (Knight and Dick, 2004). Soil extracellular enzymes immobilized on soil organo-mineral complexes may not be as sensitive to toxicants as those associated with microbial cells (Nannipieri, 1994). In addition, soil enzymes are released by a great diversity of soil living biota (e.g., protozoa, plants, and the soil meso- and macrofauna), including a huge diversity of microorganisms (bacteria, fungi, algae) (Burns and Dick, 2002). By contrast, nitrification is mainly ensured by specific functional groups of bacteria or archaea, with a minor contribution of heterotrophic fungal nitrification in agricultural soils (Prosser, 2005). A lower functional redundancy in nitrifying communities may increase the sensitivity of nitrification to a stress as compared to broader-scale processes, such as enzyme activities, ensured by a wide microbial diversity (Wertz et al., 2007; Griffiths and Philippot, 2012). The underlying activities of functional microbial groups involving in N-cycling were already reported to be more sensitive to pesticides than the soil enzymes or other microbial activities related to C-cycling (Crouzet et al., 2016; Karas et al., 2018; Rose et al., 2018).

Finally, the soil biological indexes used to summarize the overall impact of pesticides on microbial activity yielded two different analyses. On the one hand, the GMean index increased along with the increase of fungicide concentrations, highlighting that global microbial activity increased. On the other hand, the SQI decreased along with the increase in fungicide concentrations, highlighting a modification in microbial activity under pesticide pressure (increase and/or decrease). This decrease of the SQI indicates that pesticides induce a disturbance of the soil system, which was buffered in the presence of earthworms in the microcosms.

## Author Contributions

All authors conceived and designed the study. SB, AB, and OC performed the experiments, samplings, and measures on earthworms and nitrification. NC and VG performed the analysis of soil enzymatic activities. SB and OC carried out the data analysis and wrote the manuscript. All authors contributed to the review and approved the final version of the manuscript.

## Conflict of Interest Statement

The authors declare that the research was conducted in the absence of any commercial or financial relationships that could be construed as a potential conflict of interest.
